# Characterization of a Sea Buckthorn Extract and Its Effect on Free and Encapsulated *Lactobacillus casei*

**DOI:** 10.3390/ijms18122513

**Published:** 2017-11-24

**Authors:** Oana Lelia Pop, Francisc Vasile Dulf, Lucian Cuibus, Marta Castro-Giráldez, Pedro J. Fito, Dan Cristian Vodnar, Cristina Coman, Carmen Socaciu, Ramona Suharoschi

**Affiliations:** 1 Faculty of Food Science and Technology, University of Agricultural Sciences and Veterinary Medicine, Calea Mănăștur 3-5, 400372 Cluj-Napoca, Romania; oana.pop@usamvcluj.ro (O.L.P.); lucian.cuibus@usamvcluj.ro (L.C.); dan.vodnar@usamvcluj.ro (D.C.V.); cristina.coman@usamvcluj.ro (C.C.); socaciucarmen@gmail.com (C.S.); 2 Department of Environmental and Plant Protection, University of Agricultural Sciences and Veterinary Medicine, Calea Mănăștur 3-5, 400372 Cluj-Napoca, Romania; francisc_dulf@yahoo.com; 3 Instituto Universitario de Ingeniería de Alimentos para el Desarrollo, Universidad Politécnica de Valencia, Camino de Vera s/n, 46022 Valencia, Spain; marcasgi@hotmail.com (M.C.-G.); pedfisu@tal.upv.es (P.J.F.)

**Keywords:** *Lactobacillus casei*, heat treatment, gastrointestinal passage, sea buckthorn, encapsulation

## Abstract

Probiotics are bacteria that can provide health benefits to consumers and are suitable to be added to a variety of foods. In this research, viability of immobilized *Lactobacillus casei* in alginate with or without sea buckthorn lipid extract were studied during heat treatment and with an in vitro gastrointestinal model. The characterization of the lipid extract was also done using the UV-Vis spectrometry (UV-Vis), high-performance liquid chromatography photodiode array detection method (HPLC-PDA), gas chromatography coupled with mass spectrometry (GS-MS) and Cryo scanning electron microscopy (Cryo-SEM). During heat treatment, the entrapped probiotic cells proved high viability (>6 CFU log/g), even at temperatures above 50 °C. The rich in monounsaturated fatty acids sea buckthorn fraction improved the in vitro digestion passage regarding the probiotic viability. The survival of the probiotic cells was 15% higher after 2 h in the acidic medium of the simulated gastric fluid in the sample where *L. casei* was encapsulated with the sea buckthorn extract compared with the samples where no extract was added. Thus, this approach may be effective for the future development of probiotic-supplemented foods as foods with health welfare for the consumers.

## 1. Introduction

Lately, consumer interest in probiotic ingestion has increased as well as the preoccupation of cell viability at eating time. The most known and utilized products containing probiotics are mainly fermented dairy foods [1].

Probiotics are defined as live microorganisms which confer a beneficial health effect to the host when ingested in adequate amounts [2]. Species such as *Lactobacillus*, *Bifidobacteria* and *Saccharomyces* are the most utilized probiotic strains [3]. *Lactobacillus casei*, for example, was shown to improve or prevent diseases such as intestinal disorders [3,4], urogenital infections [5], obesity [6], cardiovascular diseases [7], and dental and liver diseases [8,9,10], and to generally support the immune system [11].

Many papers debate the effect of different prebiotics (food for probiotic cells) on the microflora but the association of probiotic cells with other functional elements is still quite limited.

Sea buckthorn popularity has increased due to its potential as a functional food and nutraceutical [12,13]. The lipid fraction of sea buckthorn represents a potential source monounsaturated fatty acids and carotenoids, mainly β-carotene, and can be used as food additive.

Carotenoids have an important photochemical and physiological role in flora, fauna and microorganisms [14]. In humans, carotenoids are considered to be responsible for the prevention of cardiovascular diseases, age-related degeneration and carcino-genesis due to the antioxidative function [15]. This antioxidative function is the one that protects microorganisms, in our case probiotics, from unfavorable environment conditions [16]. To our knowledge, this is the first research that associates and investigates the probiotic cell viability in the presence of a lipid fraction.

The effectiveness of probiotics intake, with all the claimed health benefits, requires ingestion of a sufficient amount of colony forming units (CFU) [17]. However, most ingested probiotic cells do not survive when passing through the gastric juice and decrease in number during storage or unfriendly environmental conditions [18,19]. A highly efficient method to alleviate these problems is cell encapsulation [20,21,22]. This versatile technology has been utilized in various fields with remarkable results.

Therefore, the present study aims to evaluate the influence of a lipid fraction of sea buckthorn on the viability of the free and immobilized *Lactobacillus casei* ATCC 393 during heat treatment and in vitro gastrointestinal model. The characterization of the lipid extract was also done using UV-Vis spectrometry, HPLC-PDA, GS-MS and Cryo-SEM. To our knowledge, this is the first study that associates and investigates the probiotic viability in the presence of a lipid fraction of sea buckthorn extract.

## 2. Results

### 2.1. Sea Buckthorn Lipid Fraction: Characterization

#### 2.1.1. HPLC-PDA and UV-VIS Spectroscopy Analysis

The β-carotene was expressed as an area percent resulted from the HPLC-PDA chromatogram. According to the calibration curve made using β-carotene standard, the total content of β-carotene from the lipid fraction of sea buckthorn is 11% (approximately 5 mg/100 g) from the total content of carotenoids. The total content of carotenoids determined using the absorbance value at 450 nm from the UV-VIS spectra was 44.7 mg/100 g. The chromatogram revealed, next to β-carotene, other free carotenoids, such as lutein, zeaxanthin, β-cryptoxanthin, α-carotene, and γ-carotene.

#### 2.1.2. GS-MS Analysis of FAMES

Table 1 and Table 2 show the identified fatty acids from the sea buckthorn lipid fraction as free and extracted from the monodisperse capsules fractions. GC-MS chromatograms revealed 19 fatty acids, including saturated fatty acids (SFA), monounsaturated fatty acids (MUFAs) and polyunsaturated fatty acids (PUFAs). The unpaired *t*-test showed that the percentage of fatty acids in the two forms of the sea buckthorn was not statistically different, proving a high encapsulation yield. Major fatty acids found in the lipid fraction of sea buckthorn are oleic (C18:1n-9), palmitoleic (C16:1n-7) (monounsaturated) and palmitic (C16:0) (saturated) acids. The fatty acids composition (Table 2) showed: MUFAs > PUFAs, MUFAs > SFAs. Furthermore, the analyzed lipid fraction was characterized by low level of PUFAs/SFAs and a good level of n-6/n-3 PUFAs, as a result of the dominants (>61%) of MUFAs. The recommended essential fatty acid balance (n-6/n-3 PUFAs) reported in literature is between 1:1 and 4:1 [23,24].

#### 2.1.3. Low-Temperature Scanning Electron Microscopy (Cryo-SEM)

**Figure 1:**
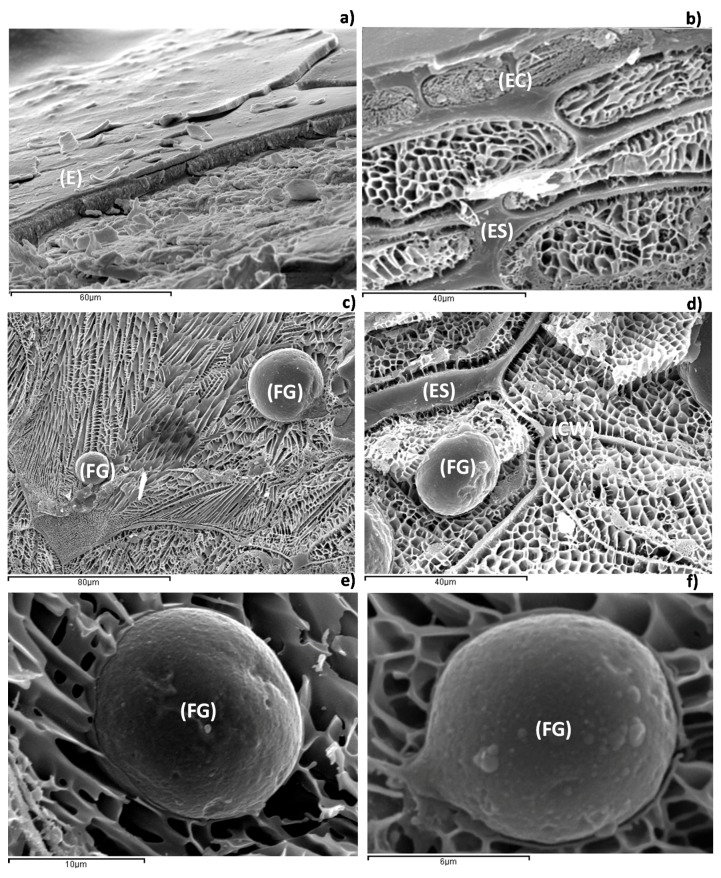
Micrographies of fresh sea buckthorn: (**a**) epidermic tissue at 1000×; (**b**) epidermic tissue at 1500×; (**c**) parenchymatic tissue at 750×; (**d**) parenchymatic tissue at 1500×; (**e**) fat globules detail at 5000×; and (**f**) fat globules detail at 10,000×. E, Epidermic tissue; EC, Epidermic cells; ES, Extracellular space; FG, Fat globules; and CW, Cell wall.

In Figure 1, it is possible to observe that the sea buckthorn presents barriers for oil extraction by mechanical extraction techniques. In Figure 1a, the epidermal tissue is observed, and Figure 1b shows the same tissue in more detail. In both figures, it is observed that the cover of the sea buckthorn has a high cell compaction. In the epidermal tissue and in the tissue immediately below (hypodermis), the presence of fat globules is not observed. The accumulation of fat globules appears in the parenchymatic tissue, which is formed by polygonal cells [25] (Figure 1c,d). In both figures, a high concentration of fat globules within the cell is shown. In Figure 1c, two fat globules, 36 µm and 15 µm in diameter, are highlighted. In Figure 1d, a fat globule of 20 µm in diameter is observed. Finally, in Figure 1e,f, lipidic globules of 15 µm and 8 µm in diameter, respectively, are shown in detail. The analysis of the parenchymatic tissue shows lipid globules of different sizes, being on average between 6 and 45 µm in diameter. The high accumulation of fat globules in the parenchymatic tissue, away from the epidermal tissue, denotes that the mechanical extraction is the most suitable method for obtaining the lipid phase of the medium.

### 2.2. Probiotic Cell Encapsulation

In this study, the probiotic-containing capsules production registered an average production of 9.48 g/min material. In the case of the capsules containing the sea buckthorn lipid fraction, the production was not significantly influenced in terms of quantity (9.51 g/min).

### 2.3. Capsules Characterization. Entrapment Efficiency

Table 3 shows size results for the two types of capsules and encapsulation yields. The yield entrapment in sodium alginate based capsules can be influenced by the capsule size, the hardening time in calcium chloride, the concentration of cells and the solution concentration [26,27]. It was observed, according to the calculated sphericity factor (SF), that the capsules with sea buckthorn had a nicer, rounder shape (SF = 0.01 ± 0.02, value is mean (*n* = 3) ± standard deviation) than the ones without sea buckthorn (SF = 0.05 ± 0.01, value is mean (*n* = 3) ± standard deviation).

### 2.4. Influence of Heat Treatment on the Probiotic Cells Viability

Our experiments involved the determination of the heat resistance of the probiotic *L. casei* in the yogurt medium, with respect to the presence of sea buckthorn lipid fraction and encapsulation. As can be observed in Table 4, the temperature of the yogurt changed depending on the microwave power and the treatment time. The temperature means showed no significant differences between the samples with free and encapsulated *L. casei*, under the same treatment. As expected, the highest temperatures were observed for the sample treated 120 s at 850 W (~65 °C).

After an analyzing the data presented in Table 4, it can be stated that highest CFU log/g is in the sample YLSE (encapsulated with sea buckthorn lipid fraction) heated at 40 °C.

Viable counts in the samples were reduced with about 25 CFU log/g when the temperature reached 55 °C, compared with the highest CFU log/g count. All the viability measurements were expressed in CFU log/g. A trend that express a protective effect of encapsulation with sea buckthorn lipid extract was observed in the sample when heated at approximately 65 °C (Table 4). For the sample where sea buckthorn was used, the no-encapsulated sample counts were 26% lower than the encapsulated ones, at the highest temperature. Meanwhile, the decrease of viability in the samples where free and encapsulated *L. casei* were tested at the highest temperature was 23% lower. These facts show that encapsulation is a technique that efficiently protects the probiotic cells, maximizing its effect when used in combination with sea buckthorn lipid fraction addition.

### 2.5. In Vitro Gastrointestinal Model Assay

**Figure 2:**
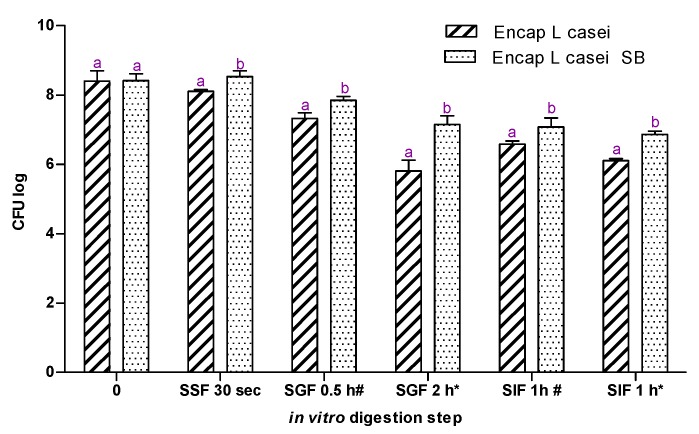
The viability of *L. casei* encapsulated with or without sea buckthorn lipid fraction, after the in vitro static digestion assay. SSF, simulated saliva fluid; SGF, simulated gastric fluid; SIF, simulated intestinal fluid; #, the sample treated 0.5 h in SGF; *, the sample treated 2 h in SGF; a, b, different letters in the same group mean significant difference (*p* < 0.05, *n* = 3 × 3).

For the in vitro digestion assay, two samples were used: probiotic loaded capsules with and without sea buckthorn extract (Figure 2). At the end of the assay, the viability of immobilized probiotic cells decreased by approximately 1.5 CFU log in the sample where no extract was used. Meanwhile, in the sample where sea buckthorn lipid extraction has been utilized, no loss of cell viability was observed. The simulated salivary fluid (SF) showed no influence regarding the viability of the probiotic cells: no significant changes were registered compared with the starting point. Viability of the probiotic cells was 15% higher after 2 h in the acidic media in the sample where *L. casei* was encapsulated with sea buckthorn extraction compared with the other sample. When a favorable pH was fulfilled, in the simulated intestinal fluid (SIF), the cells entrapped with the lipid fraction had the capacity to increase their number. Thus, the lipid extract did offer protection during in vitro gastrointestinal model and also sustained the growth of *L. casei*.

## 3. Discussion

In the present study, we have evaluated the influence of a lipid fraction of sea buckthorn on the viability of the free and alginate-encapsulated *Lactobacillus casei* ATCC 393 during the heat treatment and in vitro gastrointestinal model. Prior to utilization, the sea buckthorn lipid extract was characterized.

Several studies have reported that sea buckthorn is rich in carotenoids, which were shown to have prophylactic and therapeutic effects towards human’s health, in particular, prevention of cardiovascular diseases and cancer [28,29]. Likewise, these bioactive molecules are applied to add value and improve the nutritional role and functionality of numerous foods; on this aspect, this study widens carotenoids effects with respect to probiotics. The resulted carotenoid content in the sea buckthorn lipid fraction utilized in this work was in agreement with other studies [30]. Lu et al. [31] described the carotenoids behavior at different temperature treatments to the formation of an aggregate. They link the increase of temperature from 15 to ~50 °C to the increase of bioavailability of carotenoids due to the formation of J-aggregate (head-to-tail). This kind of aggregate can explain the rounder shape of the capsules when sea buckthorn lipid fraction is added. The trend that was reported before and discussed further by other works [32].

The composition of lipid fraction of the sea buckthorn is considered to further influence the probiotic viability. Recent studies [16,33] correlate the viability of the probiotics with new types of materials, such as sea buckthorn mucilage or omega-3 oil. Analyses and reports of the lipid fraction of the sea buckthorn are very scattered. The most analyzed ones are the fruits and derivate products (juice) [30,34,35,36] or the seed oil [12,13]. Compared to lipid fraction of the seed, rich in essential fatty acids, such as linoleic acid (C18:2, n-6) and α-linoleic (C18:3, n-3), the fruit lipid fraction contains high levels of saturated (palmitic (C16:0)), and monounsaturated (palmitoleic (C16:1n-7)) acids. The saturated fatty acids represent around 81% of the total fatty acids from this fraction. The lipid fraction is rich in palmitic acid and in monounsaturated fatty acids, namely palmitoleic (16:1) and oleic + vaccenic acids (18:1), which represented together around 91% of total fatty acids.

The lipid fraction of sea buckthorn represents a potential source of carotenoids, mainly β-carotene and fatty acids. It can be used as food additive, cosmetic ingredient, nutraceutical and, according to this study, as a protective source for *L. casei*. It is reported that for *L. casei* the metabolization of certain foods is more difficult than other strains [37]. Thus, further work can be conducted to understand better the protection mechanism of the sea buckthorn lipid fraction on different probiotic strains.

The presence of the sea buckthorn lipid fraction did not influence the capsules production concerning quantity. Instead, the shape and the size of the capsules were influenced, a trend also seen in other studies [38,39]. Large scale production of probiotic alginate capsules is quite scarce discussed in the literature. A production of approximately 9.51 g of capsules per minute, described in our study, is equivalent to little more than 0.5 kg per hour. That quantity can be utilized in almost 600 serving sizes of yogurt for example. The addition of the sea buckthorn to the alginate probiotic solution (slurry) made it creamier due to the extraction texture. This texture allows the utilization of lower concentration of alginate in the encapsulation solution, resulting in lower production costs. In our study, 1.5% alginate solution was used, but several other studies report higher concentrations [32,40,41].

It is well-known that the size of the formed capsules is influenced by the nozzle size, slurry viscosity and working parameters (frequency, flow rate) [27]. Our entrapment of probiotics in alginate matrix showed an encapsulation yield close to 100%. Utilization of alginate for the encapsulation has valuable advantages, such as high encapsulation yield [40], resistance to stomach acidity [37], controlled release in the intestinal media for colonization and biocompatibility of the polymer [41]. For the samples where the sea buckthorn lipid fraction was utilized, a 2% higher yield was registered. Other studies reported that the presence of different additives, such as sea buckthorn mucilage, lucerne green juice, potato starch, etc. positively affected not only the viability of probiotics but also the encapsulation yield [16,37,38,40].

The reduction in the CFU log pattern of the microwave treated probiotic cells was investigated using probiotic cell suspension or encapsulated probiotic cells in yogurt. The viable counts in the samples were found to diminish relative to an increase in a microwave heating temperature after a certain point (above 55 °C). For all samples, the temperature increased proportionally to the time of exposure to the microwave radiation and to the microwave power intensity, behavior reported by the Woo et al. [42]. According to our results, the addition of the lipid extract increased the viability of probiotic cells by almost 4%. The survival of microorganisms during heat treatment is influenced by several factors. One of the main factors that vary from one *Lactobacillus* spp. to another is the potential of the strain to resist at high temperatures [43,44,45,46]. Gunenc et al. [16] reported an increase of 6% in the case where whole sea buckthorn was utilized with a *Lactobacillus* strain during storage over 14 days in comparison with the sample where no sea buckthorn was added. In our study, based on two-way ANOVA analysis, all viable and active counts in the samples where no encapsulation was used were significantly lower than the samples with encapsulated probiotics. We observed that the temperature was ensuring an activation of the viability of the started cultures with approximately 50% in the plain yogurt (with no added probiotic-Y) to a certain temperature (40 °C). In the samples with added *L. casei*, the temperature induced 8% (YL, yogurt with *L. casei*) and 18% (YLS, yogurt with *L. casei* and sea buckthorn extract) increases in viability when the lipid extract was added. Temperatures above 50 °C caused a decrease of viable free probiotic cells. Comparing the encapsulated samples, the sample YLE (yogurt with encapsulated *L. casei*) registered approximately 50% loss in viability when it reached the highest temperature (64.5 °C). Meanwhile, the sample YLSE (yogurt with encapsulated *L. casei* and sea buckthorn extract) registered almost 10% higher loss, but the number of viable probiotics did not decrease below 5.5 CFU log.

After the in vitro static digestion assay, we can conclude that the presence of sea buckthorn lipid extract positively influenced the viability of the probiotic cells. No significant difference could be observed after the treatment in the SSF (simulated salivary fluid), since the samples remain for only 30 s in this media with a neutral pH. Most of the studies where the behavior of probiotics (free and encapsulated, with or without additives) is tested do not investigate the oral digestion phase. The short time of the food spends in the mouth may be the reason for this fact. Our results, which sustain that sea buckthorn lipid extract protects the entrapped probiotics in vitro gastrointestinal model assay, are in line with a study conducted by Gunenc et al. [16]. This study investigated the presence of whole fruits and purified mucilage of sea buckthorn and their influence on probiotic cell viability (*L. acidophilus*) during 28 days of storage at 4 °C and simulated gastrointestinal passage. In all of the samples where the sea buckthorn source was added, a significantly higher number of probiotic cells were counted after 28 days. The aforementioned study does not discuss the change in taste of the yogurt, despite the fact that is considered to be an important decision factor regarding the consumption, especially for children [47]. This element taste determined the use of only encapsulated samples. Another study conducted by Darjani [48] has investigated the role of inulin on *L. casei* during exposure to simulated gastrointestinal conditions. This study emphasized the effectiveness of encapsulation and even more of the presence of a supplement adding, which improved the probiotic cell viability with approximately 0.5 CFU log compared with the sample where adding was used. In our study, the influence of the lipid extract presence was more visible in the samples that were incubated 2 h in SGF. After the low pH treatment, in the samples where no extract was used, the lost in viability was approximately 0.5 CFU log higher than in the other sample. The results suggest that co-encapsulation of *L. casei* with a sea buckthorn lipid extract constitutes an alternative for maintaining the probiotic viability during heat treatment and in vitro static digestion assay, ensuring also valuable fatty acids and highly bioavailable carotenoids.

## 4. Materials and Methods

### 4.1. Sea Buckthorn (Hippophae rhamnoides) Lipid Fraction: Obtaining and Characterization

Lipid fraction from fresh sea buckthorn was obtained from smashed and centrifuged berries collected from Cluj, Romania, and stored at 4 °C before use. Different analyses were conducted on the sea buckthorn lipid fraction such as UV-VIS spectrometry, HPLC, and GC-MS. Moreover, the microstructure of fresh sea buckthorn was analyzed by Cryo-SEM.

#### 4.1.1. HPLC-PDA and UV-VIS Analysis

Total carotenoids were extracted from the freshly obtained puree (0.5 g) using methanol:ethyl acetate:petroleum ether (1:1:1, *v/v/v*). The mixture was mixed for 1 min, kept 15 min in an ultrasonic bath and centrifuged for 10 min at 2000 RPM. The supernatant was collected and the extraction procedure was repeated two more times. The total volume of the final extraction was 5.5 mL.

The qualitative analysis of the carotenoids extract was conducted using an HPLC coupled PDA detector. The quantity of β-carotene was determined using a compound standard and a calibration curve. A Shimadzu HPLC-PDA chromatograph was used employing a LiChrosorb RP 18 column. For the analysis, two mobile phases were used: solvent A—acetonitrile:water 9:1, *v*/*v* with 0.25% trimethylamine; and solvent B—ethyl acetate with 0.25% trimethylamine. The total content of β-carotene was determined using the HPLC analysis. Calibration curves for β-carotene were prepared at seven concentrations in the range 0–300 µg/mL by plotting the peak area recorded by DAD against the known concentration of the standard. The linear regression factor of the calibration curves was greater than 0.98. The total carotenoids content was determined spectrophotometrically using a Jasco V 530 spectrophotometer with a double beam. The UV-Vis spectra were registered at 300–550 nm. Carotenoids content was calculated using the absorption value at 450 nm according to the formula:

X = A × Y × 1000 × dilution/2500 × 100(1)

where X is the weight of carotenoids in the sample (mg); A is the absorbance (λ max = 450 nm); Y is the volume of the sample (mL); 2500 is the molar absorption coefficient (E_1%_) for carotenoids.

Data were processed by specific software (Shimadzu LC Solution and Spectra Manager for Windows 10/NT).

#### 4.1.2. GS-MS Analysis of FAMEs

The FAMEs (fatty acid methyl esters) were prepared using acid catalyzed transesterification of the total lipid fractions [49].

The analysis of FAMEs was achieved by capillary gas chromatography, using a PerkinElmer Clarus 600T GC-MS (PerkinElmer, Inc., Shelton, CT, USA). The column of the GC was a Supelcowax 10 (60 m × 0.25 mm i.d., 0.25 µm film thickness; Supelco Inc., Bellefonte, PA, USA) [49]. The working temperature of the oven was set at 140 °C, then increased to 220 °C at 7 °C/min, and kept for 23 min at 220 °C. The injection volume was 0.5 µL (split ratio of 1:24) and the temperature of the injector was set at 210 °C. The used carrier gas was helium, with a constant flow rate of 0.8 mL/min. Mass spectra were recorded at 70 eV and using a trap current of 100 µA with a source temperature of 150 °C. The MS was scanned at *m*/*z* 22–395 for all GC-MS experiments. The identification of fatty acids was accomplished by comparing their retention times with those of known standards and the resulting mass spectra to those in the database (NIST MS Search 2.0). The amount of each fatty acid was expressed as peak area percentage of total fatty acids.

#### 4.1.3. Low-Temperature Scanning Electron Microscopy (Cryo-SEM)

The Cryo-SEM experiments were carried out using a Jeol JSM-5410 scanning electron microscope (Jeol, Tokyo, Japan) coupled to a CryoACryostage CT-1500C unit (Oxford Instruments, Witney, UK). The instrument was operated at 15 kV, at a working distance of 15 mm and a temperature below −130 °C. For the measurements, the sample was immersed in liquid nitrogen (−210 °C) and further transferred to the Cryostage at 1 kPa where the sample was cut. The sublimation (etching) took place at −95 °C. The final point was determined by direct observation at 5 kV acceleration voltage. Prior to measurements, the sample was coated with gold in a vacuum (0.2 kPa, 3 min, ionization current 2 mA).

### 4.2. Bacterial Strains and Culture Conditions

*L. casei* ATCC 393 (ATCC) was cultivated sequentially in 10, 50, 100 and 400 mL sterile de Man, Rogosa and Sharpe (MRS) broth (Merck, Darmstadt, Germany) overnight at 37 °C. The cells were harvested by centrifugation (Eppendorf Centrifuge 5804 R, Hamburg, Germany) at 1500 RPM, 10 min at 4 °C and washed twice with 9 g/L NaCl.

### 4.3. Probiotic Cells Encapsulation

For the encapsulation of the probiotic cells, we used a laminar flow drip casting method as described in a previews work [27], but with slight modifications. For this, 15 g/L sterile sodium alginate solution was mixed with centrifuged and washed probiotic cells and dripped using a vibrational unit (Multinozzle Biotech-EncapBioSistems Inc. encapsulator) into a sterile 20 g/L calcium chloride solution, used as the hardening bath according to the experimental design. A 350 mm nozzle was used. The obtained capsules were used fresh in the further experimental work. After the encapsulation, we obtained different types of samples described in Table 5.

### 4.4. Capsules Characterization, Entrapment Efficiency and Sphericity

The theoretical and the corrected diameter of the capsules was determined using a method described previously [50]. The capsules shape was quantified using the SF, which is given by the following equation:

SF = d_max_ − d_min_/d_max_ + d_myin_ (2)

where d_max_ is the largest diameter and d_min_ is the smallest diameter perpendicular to d_max_.

For the determination of the d_max_ and d_min_, ten capsules were used and the average was calculated. The d_max_ and d_min_ were obtained using an optical microscope fitted with a calibrated micrometer scale.

The entrapment efficiency was determined according to a method previously described [51,52]:

Entrapment efficiency = (a × F/b) × 100(3)

where a is CFU log/g in the capsules, b is CFU log/g in the mixture before encapsulation and F is the sphere packing factor [53], which was considered to be 0.70 for all calculations. The obtained capsules were characterized regarding size, surface and sphericity using an optical microscope.

### 4.5. Heat Treatment

*L. casei* incorporated in yogurt in free and encapsulated forms was treated under microwave heating at 100 W, 450 W and 850 W. The microwave treatment was carried out for all the samples during 10, 30, 60, 90 and 120 s, respectively, at all of the aforementioned microwave power levels. After each microwave treatment, the temperature of the samples was measured. Next, the viability of the probiotic cells from each sample was determined. The results were used to evaluate the influence of the microwave on the free and encapsulated *L. casei*, with or without sea buckthorn (lipid fraction of the puree). Survival curves at mentioned microwave power levels and temperatures (4–80 °C) were obtained. The temperature of all samples was approximately 4 °C before the microwave treatment.

### 4.6. In Vitro Gastrointestinal Model

For the in vitro digestion assay, a standardized static method described by Minekus et al. [54], with slight modifications, was used. For the oral phase, we mixed 5 g of sample (yogurt with encapsulated probiotic with or without sea buckthorn) with 3.5 mL simulated saliva fluid (SSF) made of 1.13 g/L KCl, 0.5 g/L KH_2_PO_4_, 1.14 g/L NaHCO_3_, 0.014 g/L MgCl_2_·6H_2_O and 0.006 g/L (NH_4_)_2_CO_3_. After vigorous mixing, 0.5 mL α-amylase (Sigma, Darmstadt, Germany) solution 1500 U/mL, 25 µL 0.3M CaCl_2_ and 975 µL water were added and mixed again. No mastication was mimicked, yogurt is a soft food, usually not chewed. The pH was adjusted with 1 M NaOH to a final pH of 7.

After 30 s in the SSF, 10 mL of the oral liquid was moved in 7.5 mL simulated gastric fluid (SGF) stock solution made of 0.514 g/L KCl, 0.122 g/L KH_2_PO_4_, 2.1 g/L NaHCO_3_, 2.76 g/L NaCl, 0.01 MgCl_2_(H_2_O)_6_ and 0.048 g/L (NH_4_)_2_CO_3_. To this digestion mixture, 1.6 mL porcine pepsin (Sigma, U.S.A) stock solution of 25,000 U/mL prepared in SGF, 5 µL CaCl_3_ 33.294 g/L, 0.3 mL 1M HCl to adjust the pH to 3 and 0.695 µL water were added.

After 0.5 h (*) and 2 h (#), 20 mL of the gastric chime was mixed with 11 mL of simulated intestinal fluid (SIF) made of 0.507 g/L KCl, 0.109 g/L KH_2_PO_4_, 7.140 g/L NaHCO_3_, 2.244 g/L NaCl, and 0.031 MgCl_2_(H_2_O)_6_. To this mixture, 5 mL pancreatin solution 800 U/mL made in SIF, 2.5 bile salt solution (65.371 g/L), 40 µL CaCl_2_ 33.294 g/L, 1.31 mL water and 0.14 mL 1 M NaOH were added to reach a pH of 7.

### 4.7. Probiotic Cells Viability Test

For the determination of the probiotic cells viability, samples were diluted (1:9 *w*/*v*) in 9 g/L sodium chloride solution. Aliquots of 100 µL were spread on a Petri dish with MRS agar using a sterile Drigalski spatula. After 36 h of incubation at 37 °C, the colonies were counted. For the samples with microencapsulated probiotics, the capsules were beforehand treated in sodium phosphate buffer (pH 6.5) for the release of entrapped probiotic cells.

### 4.8. Statistical Analysis

All the experiments were conducted in triplicates. The statistical evaluation was carried out using Graph Prism Version 4.0 (Graph Pad Software Inc., San Diego, CA, USA). Statistical differences among samples were estimated using Student’s *t*-test and one-way analysis of variance (ANOVA). The significance of difference was defined at the 5% level (*p* < 0.05). Calculations regarding the capsules size determination were performed using Microsoft Excel 2010.

## 5. Conclusions

In conclusion, the presence of a lipid extraction of sea buckthorn can positively influence the viability of probiotic cells during heat treatment and in vitro gastrointestinal passage. The viability of probiotic cells can be improved by approximately 12% in yogurt if only consumed between 20 and 40 °C, and by about 16% if a lipid fraction of sea buckthorn is used in the encapsulated form of the probiotics. The rich in monounsaturated fatty acids sea buckthorn fraction improved the in vitro digestion passage of alginate encapsulated *L. casei*. The survival of the probiotic cells was 15% higher after 2 h in the acidic media of the simulated gastric fluid in the sample where *L. casei* was encapsulated with the sea buckthorn extract compared with the samples where no extract was added.

## Figures and Tables

**Table 1:** Fatty acid compositions (molar % of total fatty acids) (determined by GS-MS) of total lipids from the lipid fraction of sea buckthorn (free and encapsulated).

Fatty Acids	SE Lipid Fraction		SE from Capsules	
	%	SD	%	SD
Caprylic	0.04	0.01	0.01	0.01
Capric	0.03	0.01	0.02	0.01
Lauric	0.04	0.01	0.05	0.01
Myristic	0.18	0.02	0.20	0.02
Z-11-Tetradecenoic acid	0.05	0.01	0.02	0.01
Pentadecanoic	0.07	0.02	0.06	0.01
Palmitic	26.59	1.33	27.94	1.45
7-Hexadecenoic acid	0.03	0.01	0.03	0.01
Palmitoleic	26.2	1.31	25.15	1.30
Margaric acid	0.02	0.01	0.04	0.01
Stearic	1.11	0.06	1.13	0.06
Oleic	27.73	1.40	27.83	1.35
Vaccenic	10.73	0.55	10.05	0.45
Linoleic	5.42	0.25	5.73	0.29
Alfa-linolenic	1.45	0.07	1.36	0.07
Arachidic	0.14	0.02	0.14	0.01
Gondoic	0.11	0.02	0.16	0.02
Behenic	0.02	0.01	0.03	0.01
Erucic	0.03	0.01	0.08	0.01

The values represent the mean of tree samples, analyzed in triplicate. SD, standard deviation.

**Table 2:** The composition (molar % of total fatty acids) classed in total lipids from the lipid fraction of sea buckthorn (free and encapsulated).

Fatty Acids	SE lipid Fraction	SE from Capsules
ΣSFAs	28.25 ± 1.41 ^b^_a_	29.62 ± 1.48 ^b^_a_
ΣMUFAs	64.88 ± 3.24 ^a^_a_	63.29 ± 3.16 ^a^_a_
ΣPUFAs	6.87 ± 0.34 ^c^_a_	7.09 ± 0.35 ^c^_a_
n-3 PUFA	1.45 ± 0.07 _a_	1.36 ± 0.07 _a_
n-6 PUFA	5.42 ± 0.27 _a_	5.73 ± 0.29 _a_
n-6/n-3	3.74 _a_	4.21 _a_
PUFAs/SFAs	0.24 _a_	0.24 _a_

Values represent mean ± SD of three samples analyzed individually in triplicate (*n* = 3 × 3). The means in the same column followed by different superscript letters (a, b, c) indicate significant differences (*p <* 0.05) among fatty acid classes (ANOVA “Tukey’s Multiple Comparison Test”). The means in the same row followed by different subscript letters indicate no significant differences (*p <* 0.05) among lipid classes of samples (unpaired *t*-test); SFAs, saturated fatty acids; MUFAs, monounsaturated fatty acids; PUFAs, polyunsaturated fatty acids.

**Table 3:** Size and encapsulation yield.

Microparticles	Microparticles Size (µm) (*n* = 10)	Encapsulation Yield (%) (*n* = 10)
Alginate 1.5%	1255.5 ± 12.7	96.13 ± 0.28 ^a^
Alginate 1.5% and 10% SE	1285.5 ± 1.3	98.46 ± 1.08 ^b^

The means ± SD with different letters (a, b) in the same column are significantly different (*n* = 3, *p* < 0.05). SE, sea buckthorn lipid fraction; *n*, number of measured capsules.

**Table 4:** Temperature variation in 250 mL yogurt in correlation with the time and the microwave frequency. Temperature unit is °C. Viability of probiotic cells YLE, yogurt with encapsulated *L. casei*; YLS, yogurt with added *L. casei* and 10% sea buckthorn lipid fraction; and YLSE, yogurt with encapsulated *L. casei* and 10% sea buckthorn lipid fraction after microwave treatment at: 100; 450; and 850 W. Values represent mean ± SD of three samples, analyzed individually in triplicate (*n* = 3 × 3).

Treatment Time (s)			0	10	30	60	90	120
Microwave Power/Sample								
100 W	YLS	CFU log/g	7.58 ± 0.07	6.69 ± 0.21	7.28 ± 0.14	7.26 ± 0.05	7.75 ± 0.32	8.33 ± 0.41
	YLE		7.89 ± 0.5	8.36 ± 0.12	7.32 ± 0.22	7.31 ± 0.5	7.45 ± 0.02	8.03 ± 0.14
	YLES		8.17 ± 0.21	8.53 ± 0.4	8.42 ± 0.13	8.63 ± 0.18	8.31 ± 0.5	9.05 ± 0.22
Temp °C			4 ± 0.2	5.5 ± 0.2	7.14 ± 0.2	13.2 ± 0.2	19.5 ± 0.2	23.2 ± 0.2
450 W	YLS	CFU log/g	7.47 ± 0.12	7.74 ± 0.23	7.44 ± 0.2	8.12 ± 0.4	8.66 ± 0.24	8.87 ± 0.09
	YLE		7.89 ± 0.51	7.65 ± 0.2	7.39 ± 0.12	7.36 ± 0.15	8.11 ± 0.8	8.98 ± 0.18
	YLES		7.78 ± 0.29	7.80 ± 0.02	7.62 ± 0.05	7.56 ± 0.5	7.79 ± 0.17	9.21 ± 0.13
Temp °C			4 ± 0.2	6.3 ± 0.2	12 ± 0.2	18.7 ± 0.2	39 ± 0.2	41 ± 0.2
850 W	YLS	CFU log/g	7.65 ± 0.03	7.65 ± 0.45	7.52 ± 0.32	7.77 ± 0.15	6.16 ± 0.16	3.81 ± 0.4
	YLE		7.89 ± 0.22	7.84 ± 0.41	7.68 ± 0.35	8.38 ± 0.05	6.83 ± 0.09	4.12 ± 0.12
	YLES		8.06 ± 0.19	7.89 ± 0.16	7.84 ± 0.24	8.86 ± 0.41	7.69 ± 0.32	5.56 ± 0.24
Temp °C			4 ± 0.2	11.4 ± 0.2	14.9 ± 0.2	30 ± 0.2	55 ± 0.2	64.5 ± 0.2

**Table 5:** Experimental design for evaluation of sea buckthorn extract addition effect on probiotic cell viability in different microwave treatment of inoculated yogurt.

Yogurt trials	SE*	Sample Coding
Yogurt with *L. casei* and 10% SE	10%	YLS
Yogurt with *L. casei* encapsulated	-	YLE
Yogurt with *L. casei* and 10% SE encapsulated	10%	YLSE

SE*—sea buckthorn lipid fraction.
